# Supplementary material for: The tumor microbiome as a predictor of outcomes in patients with metastatic melanoma treated with immune checkpoint inhibitors
Source: bioRxiv. 2023 May 25:2023.05.24.542123. Preprint. [Version 1] doi: 10.1101/2023.05.24.542123 (PMC10245822; doi:10.1101/2023.05.24.542123)

Supplementary Figure 1. Association of CIBERSORT cell types with the response to ICIs.

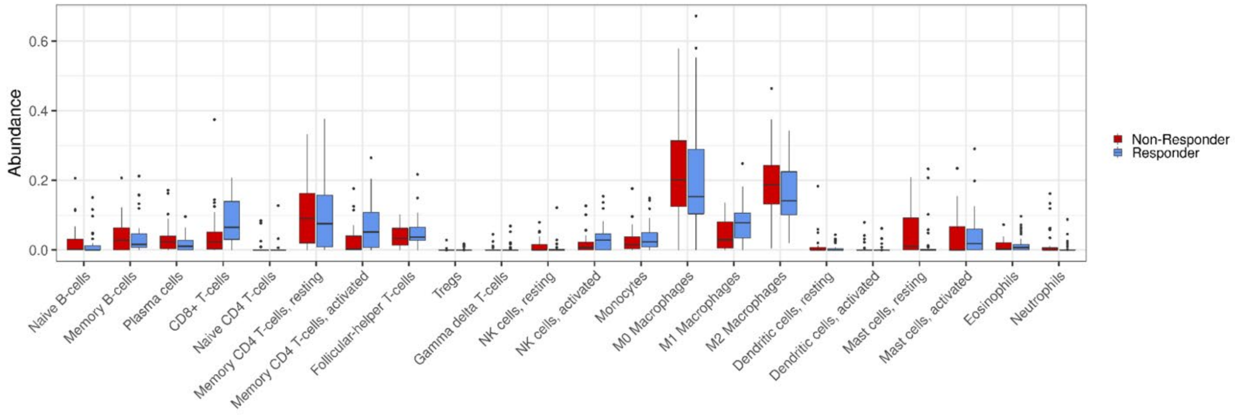

bioRxiv preprint doi: https://doi.org/10.1101/2023.05.24.542123; this version posted May 25, 2023. The copyright holder for this preprint (which was not certified by peer review) is the author/funder, who has granted bioRxiv a license to display the preprint in perpetuity. It is made available under aCC-BY 4.0 International license.

Supplementary Figure 2. 16 gene signatures where high Z-scores are associated with ICIs responsiveness in this cohort (FDR <0.05).

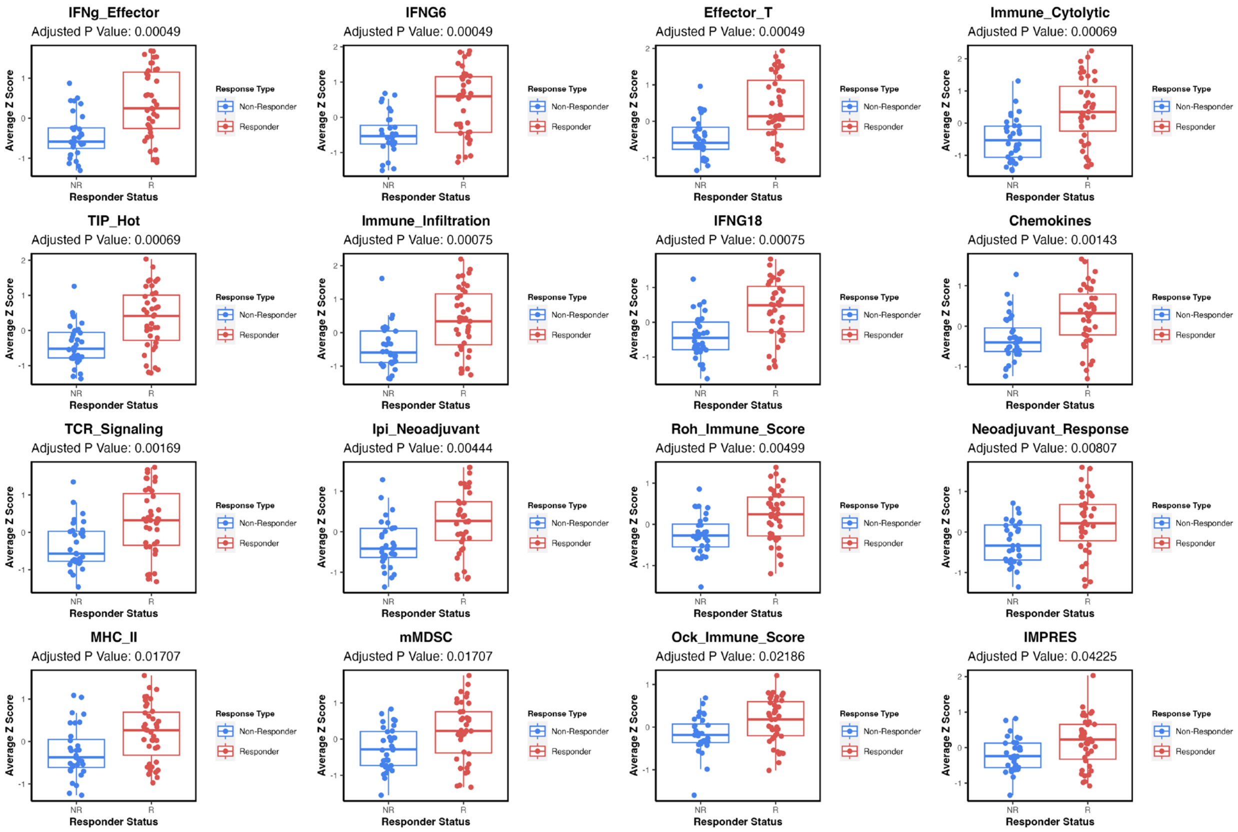

Supplementary Figure 3. Predictive values (AUROC) of the 16 gene signatures in the ORIEN cohort (IO_NOVA_Mel) and 22 other melanoma cohorts.

bioRxiv preprint doi: https://doi.org/10.1101/2023.05.24.542123; this version posted May 25, 2023. The copyright holder for this preprint (which was not certified by peer review) is the author/funder, who has granted bioRxiv a license to display the preprint in perpetuity. It is made available under aCC-BY 4.0 International license.

Signature	AUC	Mann Whitney P Value	Mann Whitney Adj. P Value
IFNg_Effector	0.78	3.00E-05	4.90E-04
IFNG6	0.78	4.00E-05	4.90E-04
Effector_T	0.77	5.00E-05	4.90E-04
Immune_Cytolytic	0.76	1.00E-04	6.90E-04
Immune_Infiltration	0.76	1.60E-04	7.50E-04
TIP_Hot	0.76	1.10E-04	6.90E-04
IFNG18	0.75	1.70E-04	7.50E-04
Chemokines	0.74	3.70E-04	0.00143
TCR_Signaling	0.74	4.90E-04	0.00169
Ipi_Neoadjuvant	0.72	0.00143	0.00444
Roh_Immune_Score	0.71	0.00177	0.00499
Neoadjuvant_Response	0.7	0.00312	0.00807
MHC_II	0.69	0.00717	0.01707
mMDSC	0.68	0.00771	0.01707
Ock_Immune_Score	0.68	0.01058	0.02186
IMPRES	0.66	0.02181	0.04225
TLS	0.62	0.0951	0.16378
MHC_I	0.6	0.14777	0.22904
gMDSC	0.57	0.35446	0.49947
DNA_Damage_Repair	0.54	0.55402	0.68699
Proliferation	0.54	0.60117	0.71678
Cell_Cycle	0.53	0.64178	0.71968
Angiogenesis	0.48	0.79612	0.79612
Hypoxia	0.48	0.75215	0.77722
Glycolysis	0.47	0.65003	0.71968
Stroma	0.47	0.71752	0.76701
WNT	0.46	0.55402	0.68699
RAS	0.45	0.44458	0.59922
Mitoscore	0.4	0.16124	0.23802
Buffa	0.39	0.10186	0.16619
MYC	0.38	0.07599	0.13857

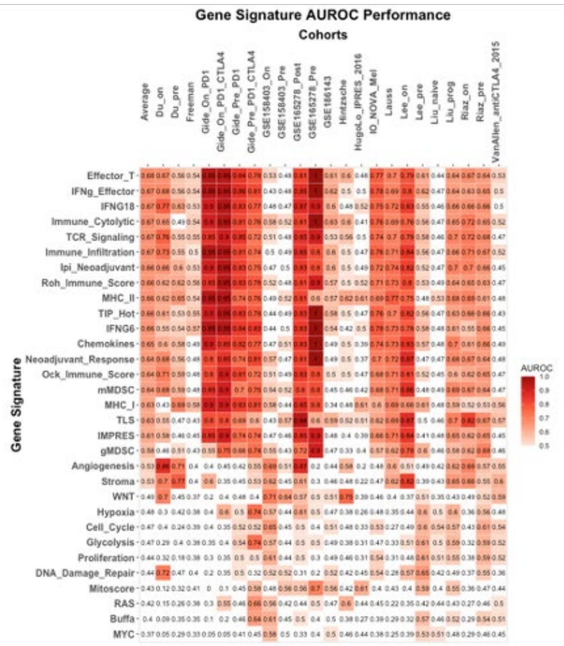

Supplement: Supplement 2 [file NIHPP2023.05.24.542123v1-supplement-2.pdf]
